# MALDI‐TOF‐MS analysis in low molecular weight serum peptidome biomarkers for NSCLC

**DOI:** 10.1002/jcla.24254

**Published:** 2022-02-25

**Authors:** Yufan Song, Xiaoyu Xu, Nana Wang, Ting Zhang, Chengjin Hu

**Affiliations:** ^1^ Departments of Laboratory Medicine The 960^th^ Hospital of the PLA Joint Logistics Support Force Jinan China

**Keywords:** biomarker, low molecular weight, matrix‐assisted laser time‐of‐flight mass spectrometry, non‐small cell lung cancer, proteomics

## Abstract

**Objects:**

Lung cancer is one of the leading causes of death from cancer in the world. Screening new serum biomarkers is important for the early detection of lung cancer. The purpose of this study was to investigate the serum peptide model between non‐small cell lung cancer (NSCLC) patients and healthy controls, as well as between paired pre‐ and postoperative NSCLC patients, and to find the low molecular weight (LMW) potential tumor markers for NSCLC.

**Methods:**

56 serum samples from NSCLC patients, 56 controls, and 20 matched pre‐ and postoperative patients were analyzed using magnetic‐bead (MB)‐based purification technique combined with MALDI‐TOF‐MS. To distinguish NSCLC from cancer‐free controls, three models were established. Finally, comparing the three groups of serum protein fingerprints, nano‐liquid chromatography–electrospray ionization tandem mass spectrometry was used to further identify the differential peptides.

**Results:**

Among the three models constructed, the GA model had the best diagnostic efficacy. Five differential peaks were screened by combining the case group, healthy controls, and postoperative group analysis, which were up‐regulated in the case group and showed a tendency to return to healthy control values after surgery. The protein matching the mass spectrometry peak m/z 2953.73 was identified as fibrinogen α chain.

**Conclusion:**

This study shows that the application of MALDI‐TOF‐MS is a promising approach for the identification of potential serum biomarkers for NSCLC, which is potentially valuable for establishing a new diagnostic method for lung cancer. In addition, we found that fibrinogen α chain may be an auxiliary diagnostic indicator for NSCLC.

## Graphical abstract

The aim of this study was to explore the differential peptides of non‐small cell lung cancer (NSCLC) based on the late‐model mass spectrometry technology of proteomics. We established the diagnostic prediction model that distinguished NSCLC samples from the healthy controls, found the potential tumor markers that can assist the early diagnosis of NSCLC, and provide new clues for the screening of high‐risk groups of NSCLC and the evaluation of postoperative efficacy.

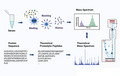

## 1 INTRODUCTION

Internationally, lung cancer continues to be the leading cause of cancer‐related deaths in men and women.^1^ Due to the insidious onset symptoms, most patients are already in the middle‐to‐late stage or have metastases when first diagnosed and have lost the best opportunity for surgical treatment. The 5 years survival rate is less than 15%,^2^ and the prognosis is poor. At present, pathological biopsy is the gold standard for the diagnosis of lung cancer, but it is an invasive procedure, which is not suitable for the early screening of high‐risk groups. Traditional imaging examination is a common method for the preliminary diagnosis of lung cancer, and the application of low‐dose spiral CT has improved the diagnosis rate of early lung cancer to some extent; however, the technique is limited by the low specificity that leads to a large number of false positives.^3^ Despite the vast potential of existing candidates and methodologies, no single lung cancer molecular biomarker is currently being used in routine clinical practice.^4^ Therefore, there is an urgent need to develop a noninvasive with high sensitivity and specificity biomarkers to promote the early diagnosis and treatment monitoring of lung cancer. Serum tumor markers can assist diagnosis, establish prognosis, and predict tumor response in a noninvasive way, which is an appropriate source for studying changes in molecular level.^5^ Tumor proteomics is a new type of molecular research that discovers biomarkers of a specific state. By integrating genomics, transcriptomics, and protein mass spectrometry bioinformatics analysis, it provides new insights in early tumor screening, therapeutic efficacy, and biological research and has gradually become one of the most attractive topics in the discovery of disease biomarkers.

LMW serum peptidome contains some unexplored archive of histological information and is expected to yield useful biomarkers for early disease detection.^6^ However, the wide range of blood protein concentrations poses challenges for proteomic analysis. As a new high‐throughput proteomics research technology, matrix‐assisted laser time‐of‐flight mass spectrometry (MALDI‐TOF‐MS) is widely used in the analysis of serum low molecular weight proteome. A purification method based on magnetic beads (MB) is used to capture a large number of LMW peptides and proteins in biological samples. Unlike other traditional proteomics techniques, MB is more precise, robust, and rapid than traditional two‐dimensional (2‐D) gel electrophoresis.^7^ MB‐based proteomics analysis platform highlights high sensitivity and repeatability,^8^ which has the characteristics of high throughput, high sensitivity, and high specificity.^9^ It plays a crucial role in screening potential protein biomarkers in serum, disease diagnosis, and efficacy prediction.

In our previous research,^10^ we used MALDI‐TOF‐MS in combination with weak cation exchange magnetic beads (WCX‐MB) to characterize serum proteomic patterns in patients with NSCLC and successfully determined differences in those patterns between NSCLC samples and controls. Based on the obtained spectra, the classification model containing the most discriminative peaks was calculated and verified with an independent test set. The differential protein mass spectra of serum samples from patients with NSCLC preoperative and postoperative were analyzed, and the differential protein between NSCLC patients and healthy controls was compared with finding the potential specific proteins, so as to provide a reliable basis for early screening, diagnosis, and prognosis monitoring of NSCLC. At the same time, nano‐liquid chromatography (nano‐LC) coupled with MALDI‐TOF‐MS was used to identify potential serum biomarkers for NSCLC, since they might provide a new insight into the multifactorial processes that occur during NSCLC tumorigenesis.

## 2 METHODS

### 2.1 Patients and sample collection

The research protocol was approved by the local institutional review board and ethics committee, and the written informed consent of each subject was obtained before the study. A total of 132 serum samples were collected from the 960 Hospital in Jinan, of which 56 were collected from NSCLC patients (33 males and 23 females), 56 from healthy volunteers as healthy control (37 males and 19 females), and 20 matched NSCLC patients (10 males and 10 females) preoperative and postoperative. The median age of the NSCLC group was 62 years (range: 25–72). The dataset of serum samples and their groups are listed in Table 1. All serum samples were randomly divided into the training group and the verification group in a ratio of 3:1. The training group included 84 serum samples (42 patients with NSCLC and 42 healthy subjects), and the verification group included 28 serum samples (14 patients with NSCLC and 14 healthy subjects). Patients in the case group were initially diagnosed as NSCLC by pathological biopsy and had not received other surgery, radiotherapy or chemotherapy, or anticancer drug treatment, and had no evidence of malignant tumor or other chronic metabolic diseases. All samples in this study were obtained by an experienced surgeon and examined by an experienced pathologist. Tumor types were identified based on microscopic observation and immunohistochemical staining, and the diagnosis of malignancy was confirmed by independent review of notes and histopathology reports. Fasting venous blood (5 ml) was collected in the vacuum tubes from all subjects and incubated at room temperature for 30 min. The samples were then centrifuged at 3000 rpm for 15 min in a cryogenic high‐speed centrifuge. The serum samples were distributed into 200 μl aliquots each and stored in Eppendorf tubes at −80°C until analysis.

**TABLE 1:** Dataset of serum samples and their groups ROC^AUC^

Characteristic	Training group(*n* = 84)			Test group(*n* = 28)		
	NSCLC(*n* = 42)	Healthy(*n* = 42)	*p*	NSCLC(*n* = 14)	Healthy(*n* = 14)	*p*
Age(year)			0.409			0.625
Median	63	61		61	59	
Range	25~72	26~75		49~69	46~75	
Gender			0.503			0.686
Male	24(57%)	27(64%)		9(64%)	10(71%)	
Female	18(43%)	15(36%)		5(36%)	4(29%)	
Smoking						
Yes	26(62%)	24(57%)	0.657	9(64%)	8(57%)	0.699
No	16(38%)	18(43%)		5(36%)	6(43%)	
Pathological						
Adeno	29(69%)	—	—	11(79%)	—	—
Squamous	12(29%)	—	—	3(21%)	—	—
Else	1(2%)	—	—	0	—	—
Staging						
Ⅰ	7(17%)	—	—	2(15%)	—	—
Ⅱ	8(19%)	—	—	3(21%)	—	—
Ⅲ	12(28%)	—	—	3(21%)	—	—
Ⅳ	15(36%)	—	—	6(43%)	—	—

### 2.2 Separation of polypeptides by magnetic beads

According to the manufacturer's standard procedure (Bruker Daltonics), MB‐WCX (Bruker Daltonics,) was used to separate peptides/proteins from all serum samples. First, combine the magnetic beads with the protein/peptide: 10 μl MB‐WCX with 10 μl binding buffer, and 5 μl serum sample was added and mixed to be tested. Then, the Eppendorf tube was put into the magnetic separator for 1 min, and the supernatant was discarded. The second step was to wash the high‐abundance protein bound on the magnetic beads. Added 100 μl buffer solution to the Eppendorf tube and mixed it evenly, the magnetic separator was allowed to stand for 1 min, then pipette absorbed the supernatant and discarded it, and the washing step was repeated for 3 times. The final step was to elute the protein bound to the magnetic beads: 5 μl elution buffer was added to the Eppendorf tube, put it in a magnetic separator and stood for 2 min. The clear supernatant was collected in a fresh tube and mixed it with 5 μl of stabilization buffer.

### 2.3 MS detection and analysis

To prepare the MALDI target, 1 μl eluted sample was spotted onto the MTP target plate (Bruker Daltonics), dried naturally at room temperature, 1 μl CHCA matrix solution (6 mg/ml in 4% trifluoroacetic acid/50% acetonitrile) was absorbed and covered on the dry sample site following by 1:1 ratio dry point method, and then, the target point was air‐dried (cocrystallization). Flexcontrol version 3.4 (Bruker Daltonics, Germany) was used with the following settings: MS scanning range: 1‐10kda, spectral laser intensity: 75%, the MS analysis voltage of ion source 1, and ion source 2 in the positive ion linear mode was 19.6 kV and 18.3 kV, respectively, and the prism voltage was 6.9kV. Prior to formal test analysis, the instrument was calibrated using Protein Calibration Standard I and II (Bruker Daltonics,). All tests were performed in a blind manner. In addition, in order to evaluate the repeatability and overall stability of the mass spectrometer, 30 serum samples from controls were randomly selected and mixed into quality control samples for internal test.

### 2.4 LC‐ESI‐MS/MS peptide identification

EASY‐nLC1200 (Thermo Fisher) nano‐upgraded liquid chromatography UHPLC was used to separate the hydrolysates. The peptide solution was injected into a C18 reverse chromatographic capture column (Nano Acquity) (50μm×15cm×2μm) at a flow rate of 15 μl/min. The enriched peptides were then gradient eluted on a C18 analytical column (Nanoacquity) (75 μm × 150 mm × 3.5 μm). Mobile phase A was an aqueous solution containing 0.1% formic acid, and mobile phase B was an acetonitrile solution containing 0.1% formic acid. After chromatographic separation, mass spectrometry and tandem fragment scanning were performed. The scanning range was 350–4500 m/z, spray voltage 2.3kv, and ion source scanning temperature 320℃. By setting the "maximum Speed MS/MS" option to 2 s, the strongest ion exceeding 2000 technical thresholds can be selected for fragmentation. Using the high‐energy collision dissociation (HCD) mode, the fragmentation energies were 30%, 32%, and 36%, respectively. After scanning each precursor ion twice, the MS/MS spectrum entered the 60 s dynamic elimination process. The obtained MS/MS spectrographs were searched by UniProt Knowledgebase (UniProtKB) for peptide matching.

### 2.5 Statistical analysis

ClinProTools 3.0 (Bruker Daltonics, Germany) was used to analyze raw spectral data from serum samples, preprocessing raw spectral peaks by smoothing, denoising, baseline removal, and normalization^1^: to eliminate matrix peaks from interfering with experimental results, peaks with molecular mass <1000 Da were excluded^2^; fitting and removal of the baseline model (exponential decay with constant offset) for each spectrum^3^; integrated down sampling using a second‐order function to ensure constancy of the spectral peak width^4^; optimal linear filtering of the target peak shape to improve the signal‐to‐noise ratio (SNR), and filtering of the peaks to maintain SNR ≥5 in order to ensure the signal response value and the amount of peaks coming out^5^; to reduce the correlation between overlapping peaks, the base is removed using a moving average of the local minima; and^6^ alignment of individual spectral peaks with the average spectrum. The pre‐processed data were then visualized and statistically analyzed. Peaks were screened by Wilcoxon rank sum test or *t*‐test (according to the normality of data distribution). The significance was set at *p* < 0.05. After spectral preprocessing, ClinProTools was applied to build three diagnostic models using different algorithms. The GA model simulates the biological evolution process by natural selection and genetic mechanism of Darwin's biological evolution and is a method to search for the optimal solution by simulating the natural evolution process, applying this model to select the combination of peaks until the optimal peak group is found. SNN is a prototype‐based classification algorithm. If a set of spectra is divided into cancer group and control group, SNN tries to identify some characteristic spectra for each class, which are named as prototypes, in order to build a classification model. QC is a univariate ranking algorithm that generates a mean peak spectrum for each group, and the mean peak area and statistical data *p*‐values are stored in the model. QC sorts the peak area for each peak and calculates a weighted average of all peaks to classify.

## 3 RESULTS

### 3.1 Analysis of serum peptide spectrum

Within run, assays were performed on the quality control targets each 15 samples, from which 7 peaks with relative molecular weight of 1–10 kDa were selected (Table 2). The average coefficient of variation (CV) of repeatability in this batch was calculated to be 19.01% (ranging from 12.57% to 25.81%), and this CV range is considered acceptable for the analysis of complex biological samples.^11^

**TABLE 2:** Coefficient of variation of mass spectrum peak repetition of quality control sample

Mass(m/z)	CV(%)
1546.68	19.46
2662.09	25.28
2934.05	25.81
3242.71	14.62
3919.24	16.13
4093.55	19.19
5907.16	12.57

ClinProTools identified a total of 112 unique peptide peaks between the NSCLC patient samples and the controls. Among them, 37 protein peaks with statistical significance were screened out (*p* < 0.05). The ROC curve of the 37 different protein peaks was further analyzed the diagnostic capacity and the AUC value was obtained. 11 peptide peaks exhibited significant differences between the two sets of samples (Table 3).

**TABLE 3:** Expression of differential peak between NSCLC group and healthy controls

m/z	NSCLC	Healthy control	Expression in NSCLC	*p*‐value*
5906.73	230.83 ± 43.71	140.54 ± 32.0	↑	<0.000001
3242.63	28.31 ± 12.39	17.24 ± 8.52	↑	<0.000001
2953.73	87.70 ± 18.41	62.07 ± 13.36	↑	<0.000001
2588.76	2.59 ± 0.84	3.91 ± 1.19	↓	<0.000001
2105.66	1.56 ± 0.80	2.68 ± 1.53	↓	<0.000001
1066.65	0.99 ± 0.69	1.97 ± 1.12	↓	<0.000001
2322.86	1.49 ± 0.40	2.20 ± 0.58	↓	<0.000001
1042.69	1.03 ± 0.49	1.74 ± 0.70	↓	<0.000001
4055.47	1.10 ± 0.30	1.61 ± 0.49	↓	<0.000001
7470.31	0.44 ± 0.14	0.66 ± 0.18	↓	<0.000001
9433.09	0.27 ± 0.10	0.38 ± 0.12	↓	<0.000001

Note

*Calculated based on a *t*‐test or the Wilcoxon test

**FIGURE 1:**
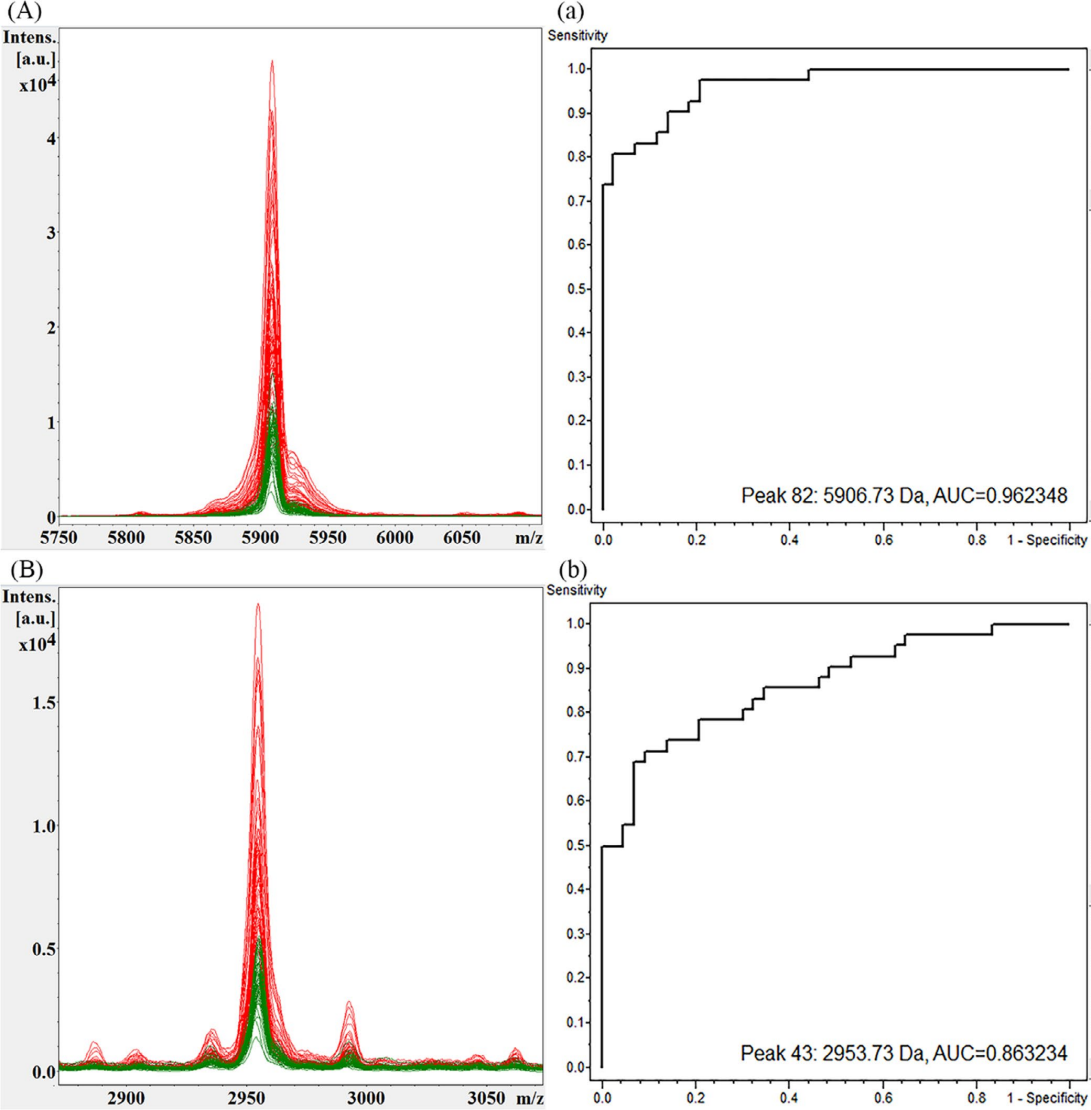
Superposition spectra and ROC curves of m/z 5906.73 and m/z 2953.73. Comparison of the spectra of peaks m/z 5906.73 and 2953.73 in two groups described above (A) (B). Receiver operating characteristic (ROC) curves for the peaks are shown with their area under the curve (AUC) values (A) (B)

**FIGURE 2:**
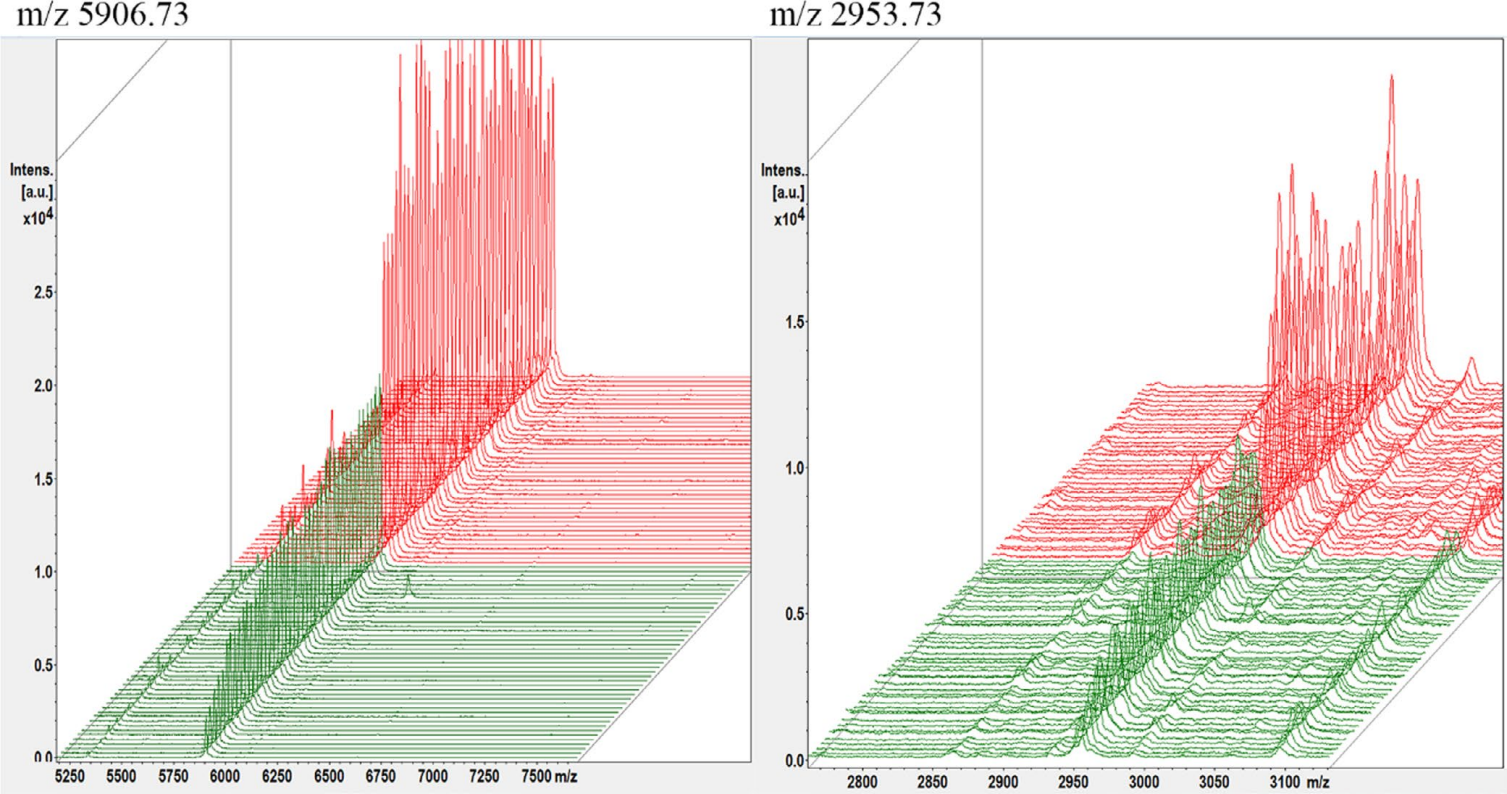
Three‐dimensional stack diagram of differential peaks m/z 5906.73 and m/z 2953.73 (red, NSCLC patients group; green, healthy control group)

**FIGURE 3:**
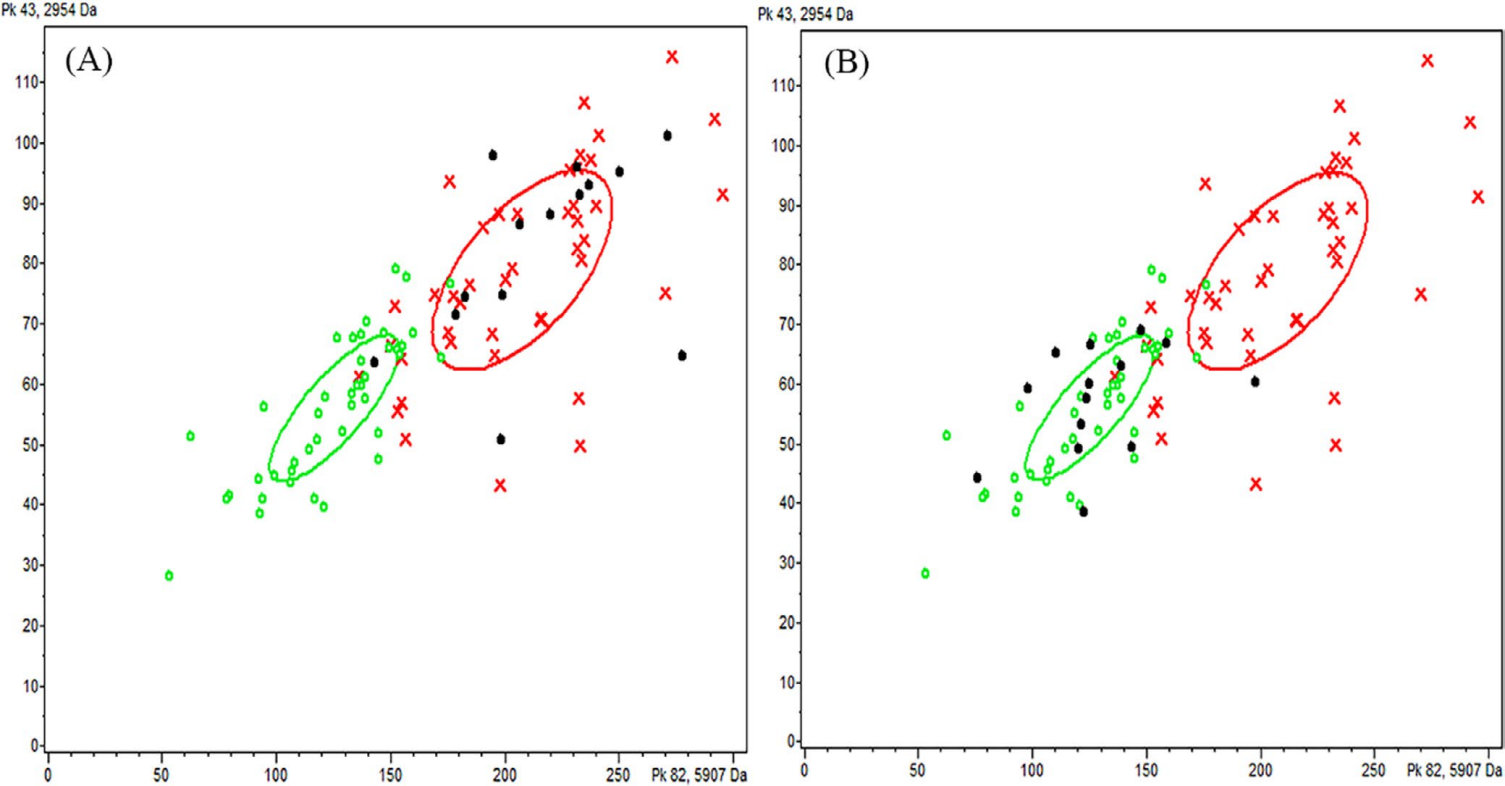
Cluster analysis of serum polypeptides of the NSCLC group (red) and control group (green). (A)Black, NSCLC patients validation group;(B)Black, controls validation group. 2D peak distribution view of peptides with m/z 5906.73 Da (*x*‐axis) and 2953.73 Da (*y*‐axis) between NSCLC group (red cross) and healthy controls (green circle) in training set by ClinProTools 3.0 software. The two circled areas represent the standard deviation of the category average of peak area

Compared with single clinical variables and biomarkers, the combination of biomarkers can improve the accuracy of prediction. In previous studies, utilizing spectral data from NSCLC and healthy controls, three mathematical algorithms of QC, SNN, and GA were used to establish the diagnosis and prediction model of NSCLC (Table 4). After comprehensively comparing the diagnostic performance of the 3 models, it was found that the GA model has the best diagnostic performance. In this model, the mass spectrum peaks m/z 5906.73 and m/z 2953.73 showed the most significant differences, both of which were up‐regulated in the NSCLC group (Figure 1). 3D spectra of the two peaks were obtained by stakage, indicating that the expressions of the difference peaks m/z 5906.73 and m/z 2953.73 were significantly increased in the NSCLC group (Figure 2). In the component analysis, a bivariate plot of NSCLC patients (red) and healthy controls (green) showed no overlapping regions between the two groups (Figure 3).

**TABLE 4:** External validation results of the diagnostic model. (%)

Model	Recognition capability	Cross‐validation	Sensitivity	Specificity	Accuracy
QC	96.43	91.80	92.90	83.30	88.50
SNN	100.00	93.84	85.70	91.70	88.50
GA	98.84	89.21	92.90	91.70	92.30

Abbreviations: GA, Genetic algorithm; SNN, Supervised Neural Network; QC, Quick Classifier.

### 3.2 Screening of preoperative and postoperative difference peaks

**FIGURE 4:**
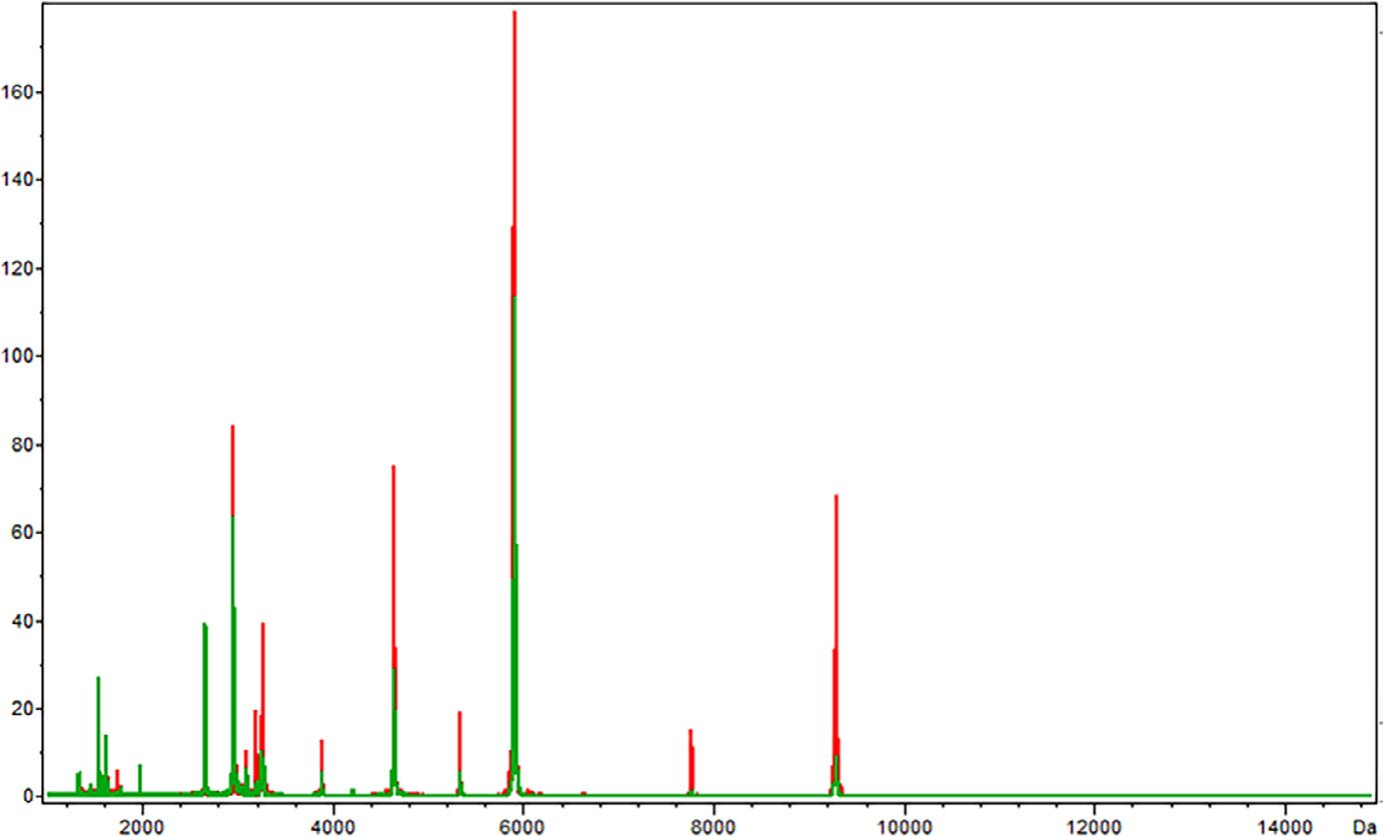
Preoperative (red) and postoperative (green) serum protein fingerprints of NSCLC

A comparative analysis of preoperative and postoperative serum samples from patients with NSCLC showed that a total of 70 different protein/peptide peaks were found between the two groups. The average spectrum of the two groups was shown in the figure (Figure 4), of which 19 were statistically significant (*p* < 0.05). In the postoperative group, 6 differentially expressed peaks were down‐regulated in the postoperative group, while the other 13 differentially expressed peaks were up‐regulated in the postoperative group (Table 5).

**TABLE 5:** Serum differential peptide peaks of preoperative and postoperative

Mass	Dave	PTTA	PWKW	PAD	Ave1	Ave2	Expression
9289.06	65.19	<0.01	<0.01	0.106	75.85	10.66	↓
5906.50	58.02	<0.01	0.036	0.374	180.21	122.19	↓
4645.07	52.26	<0.01	<0.01	0.335	84.54	32.28	↓
5922.39	44.90	<0.01	<0.01	< 0.01	16.97	61.88	↑
2961.18	33.64	<0.01	<0.01	< 0.01	11.92	45.56	↑
1547.05	24.90	0.274	<0.01	< 0.01	3.89	28.79	↑
7767.06	20.67	<0.01	<0.01	0.063	22.82	2.15	↓
1618.44	12.89	0.105	<0.01	< 0.01	4.94	17.83	↑
3242.65	10.14	0.021	0.017	0.47	23.03	12.89	↓
3280.09	6.00	0.051	<0.01	< 0.01	3.42	9.42	↑
1607.61	5.76	0.32	<0.01	< 0.01	1.5	7.26	↑
1974.49	5.31	<0.01	<0.01	< 0.01	2.47	7.79	↑
1331.43	3.62	0.047	<0.01	< 0.01	3.53	7.15	↑
3008.07	3.11	<0.01	<0.01	< 0.01	2.2	5.3	↑
3209.42	3.09	0.043	<0.01	< 0.01	2.34	5.43	↑
2953.76	2.98	<0.01	0.953	0.101	69.06	66.08	↓
1969.27	2.91	0.025	0.036	0.434	6.63	9.55	↑
1630.07	2.76	<0.01	<0.01	0.099	2.87	5.63	↑
5354.42	2.58	0.016	<0.01	< 0.01	1.35	3.92	↑

Note

Dave: maximum mean difference in peak intensity between groups; PTTA, PWKW, PAD: *p*‐values of different test methods, PTTA, PWKW, and PAD indicate the *p* values corresponding to the different tests: PTTA (*t*‐test), PWKW (Wilcoxon test), and PAD (Anderson–Darling test), respectively; Ave1 and Ave2 represent the average peak intensity of the NSCLC group (preoperative) and postoperative group, respectively.

**FIGURE 5:**
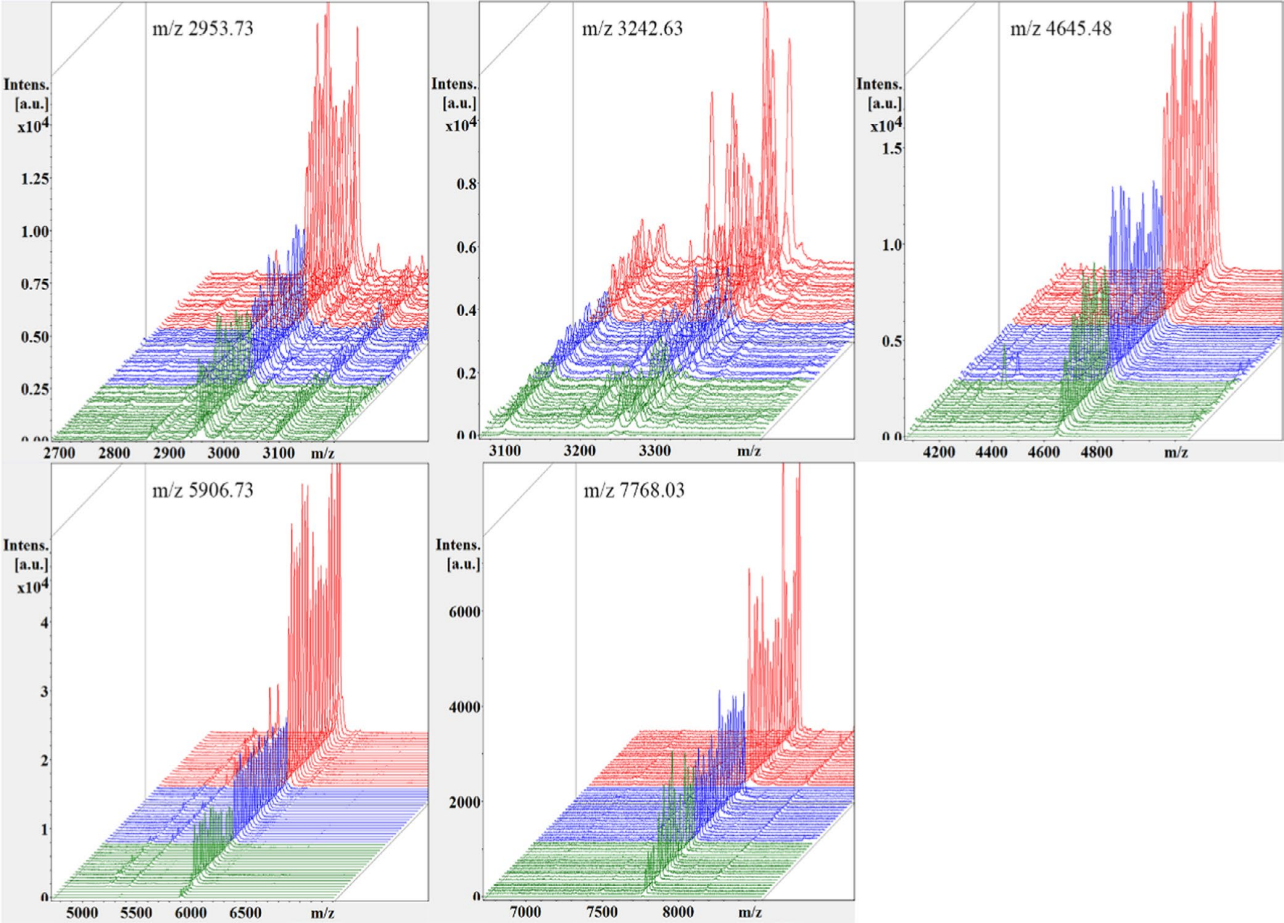
Three‐dimensional stacked spectra of the selected 5 difference peaks between the preoperative (red), postoperative (blue), and healthy control group (green)

By combining the difference peaks of the pre‐ and postoperative groups with those of the former case group and the control group, there were 5 difference peaks m/z 5906.5, 4645.07, 7767.06, 2953.76, and 3242.65 in the pre‐ and postoperative group, which were close to the peaks m/z 5906.73, 4645.48, 7768.03, 2953.73, and 3242.63 in the case group. The differences were all less than 1 Da. All the five peptide peaks were expressed up‐regulated in the NSCLC group and down‐regulated in the control and postoperative groups, and the down‐regulation trend tended to be similar to that of the control group in the postoperative group (Figure 5).

### 3.3 Identification of peptide peaks

**FIGURE 6:**
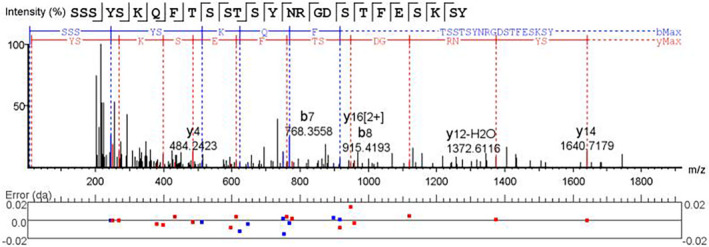
Identification of peptides present at higher levels in NSCLC patients. The peak m/z 2953.73 (sequence: SSSYSKQFTSSTSYNRGDSTFESKSY) was identified as a fragment of fibrinogen α chain

In this part of the study, 30 serum samples with high protein expression in the previous part of the study were selected and cleaved into peptides after enzymolysis by trypsin. LC‐MS/MS was used to detect the fragmented peptides and obtain the secondary mass spectrometry of the target peptide (Figure 6). Peptide peaks m/z 2953.73 had amino acid sequences of SSSYSKQFTSSTSYNRGDSTFESKSY, which corresponded to fragments of fibrinogen α chain. In contrast, the amino acid sequence corresponding to peak m/z 5906.73 could not be determined at present, and it is speculated that it may be caused by unknown modification of this peptide.

### 3.4 Bioinformatics analysis

**FIGURE 7:**
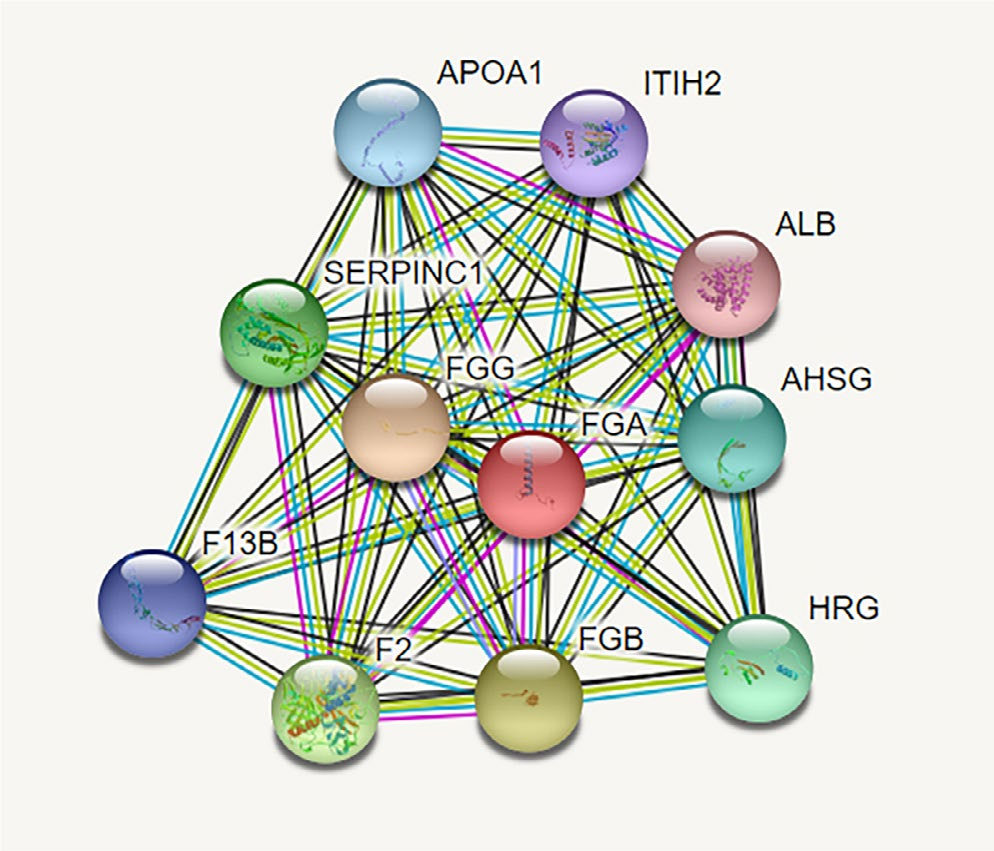
Interaction network of the proteins identified and their function‐related proteins based on STRING

**FIGURE 8:**
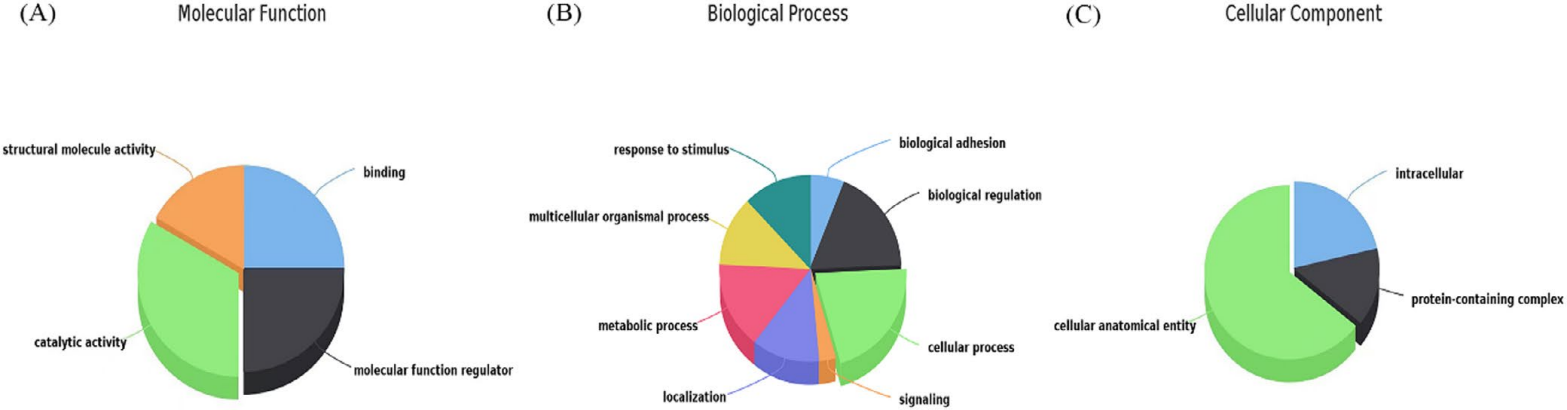
GO analysis of identified proteins based on PANTHER. (A) The distribution of identified proteins based on GO molecular functions. (B) The distribution of identified proteins based on GO biological process. (C) The distribution of identified proteins based on GO cellular components

**FIGURE 9:**
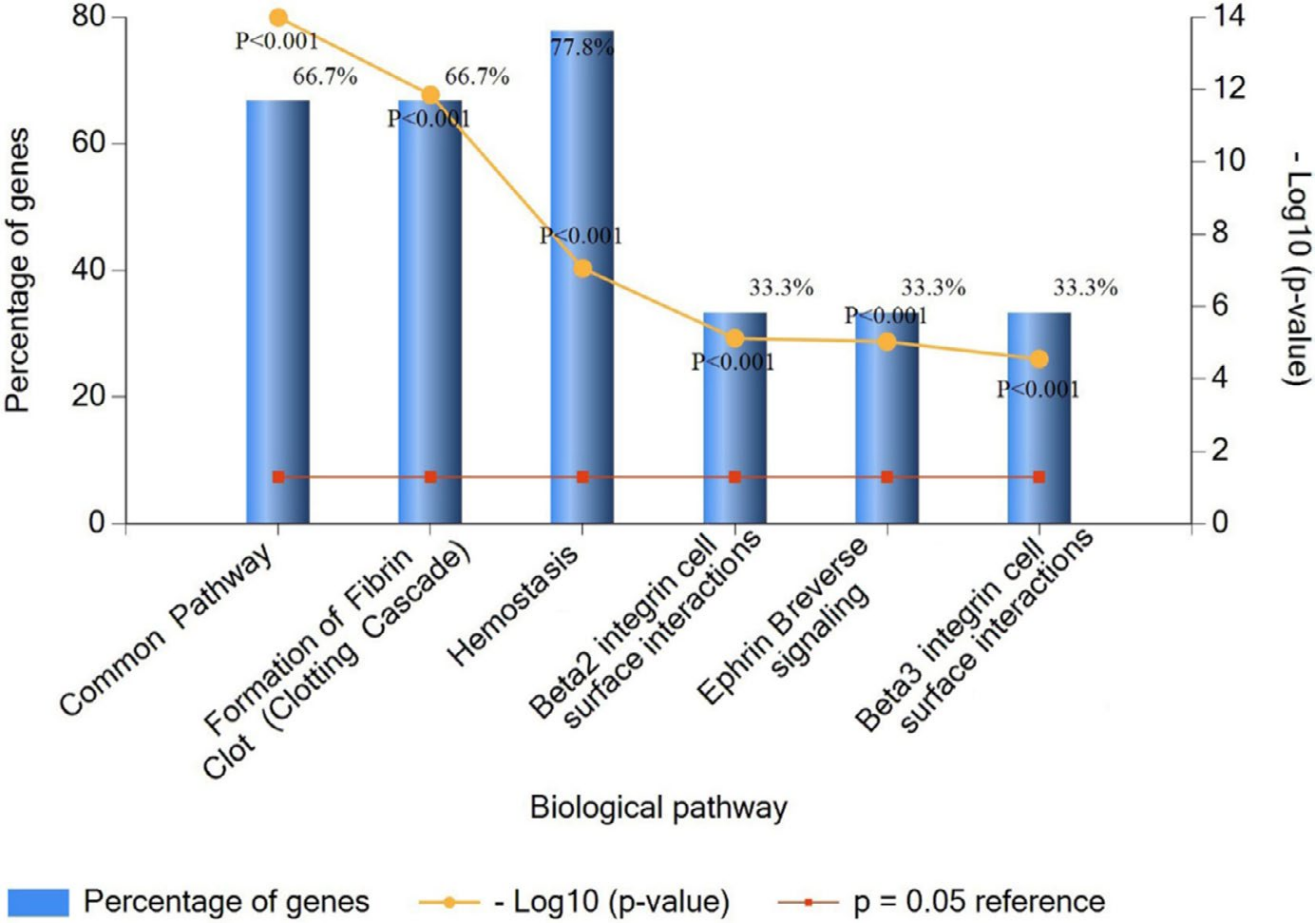
The different signaling pathway hits of these proteins

In order to further study the possible role and mechanism of fibrinogen α chain in the occurrence and development of NSCLC, string PPI network database was used to analyze and predict the related proteins (ApoA1, ITIH2, ALB, AHSG, HRG, FGB, FGG, F2, F13B, and SERPINC1) (Figure 7). Subsequently, the PANTHER classification system was used to perform GO annotation summary analysis on the molecular functions of related proteins and to initially understand the biological functions, pathways or cellular localizations of protein enrichment, and extract significantly rich biological processes, molecular functions, and pathway networks. The distribution of protein molecular function revealed that the identified proteins pattern in major functions such as binding activity (25%), catalytic activity (33%), molecular functional regulation activity (25%), and structural molecular activity (17%) (Figure 8A). The major function group (catalytic activity, 30%) was included four proteins (SERPINC1, HRG, F2, and AHSG). In addition, the identified proteins were dispersed in a variety of cellular components, including macromolecular complexes, cellular fractions, and cellular analytical entity (Figure 8C). They were involved in a wide range of biological processes, such as localization, cellular process, biological regulation, response to stimulus, biological adhesion, signal transduction, metabolism, and multicellular organismal process (Figure 8B). The biological pathway of protein was analyzed by FUNRICH to study the biochemical signal transduction pathway and metabolic pathway involved in protein, among which the most representative is coagulation cascade and fibrin clot formation (Figure 9).

## 4 DISCUSSION

Biomarkers are of great significance in lung cancer research. At present, the vast majority of lung cancer patients are diagnosed at a late stage, with a low 5 years survival rate and the most effective approach to combat tumor is still early diagnosis. The dynamic changes in serum proteome can reflect the different physiological states of the organism in the early stage of disease, and it has the advantages of noninvasive and easy to collect samples.^12^ The LMW serum proteome contains a large amount of biomarker information, but its identification is often hindered by the presence of high‐abundance proteins in serum, which makes the detection of low abundance proteins in serum extremely challenging.^13^ Therefore, reducing the complexity of sample proteins and removing high‐abundance protein levels is a crucial step in the process of characterizing serum proteins.^14^ In recent years, with the advancement of biological mass spectrometry technology, mass spectrometry has demonstrated significant analytical capabilities in the low molecular weight (1–20 kDa) signal range.^15^ MALDI‐TOF‐MS can detect a wide range of biomolecules in a concentration range near to sub‐femolar, with the characteristics of high sensitivity, high specificity, and high throughput. On the contrary, the development of tandem mass spectrometry (MS/MS) technology provides new protein sequence information for proteomics, and this powerful method can identify protein molecules without knowing its amino acid sequence, further promotes the application of the technology.^16^

In this study, we used MALDI‐TOF‐MS technology combined with magnetic‐bead preparation method to detect serum protein/peptide in patients with NSCLC and healthy controls, and 37 statistically significant difference peaks were screened out (*p* < 0.05). For the calculation model, two parameters of recognition ability and cross‐validation were determined, but due to its difficulties to select the model with the best diagnostic efficacy according to the cross‐validation and recognition ability, external validation was performed for three classification algorithms. Based on the GA algorithm model, NSCLC patients could be discriminated from healthy controls with 92.90% sensitivity and 91.70% specificity.

Through the analysis of the differential proteins/polypeptides between the preoperative and postoperative NSCLC groups and the case group, it was found that the difference peaks m/z 5906.50, 4645.07, 7767.06, 2953.76, and 3242.65 have significant statistical differences in the two parts, and the expression is up‐regulated in the NSCLC group, and down‐regulated in the control group and the postoperative group. With the tumor resection, the down‐regulation trend in the postoperative group was closer to that in the healthy control group. Therefore, it is speculated that this group of difference peaks plays an important role in the occurrence and progression of NSCLC and have a high application value in assisting the diagnosis and prognosis of NSCLC. Subsequently, we detected the post‐enzymatic peptides by LC‐ESI‐MS/MS, and UniProtKB database was used to review the database. Among the two candidate factors for potential tumor markers of NSCLC, the protein matched by the difference peak with m/z of 2953.73 is fibrinogen alpha chain.

Fibrinogen is a complex fibrous glycoprotein secreted by hepatocytes and consists of a dimeric structure of 3 different pairs of polypeptide chains α, β, and γ. It has oncogenic effects by binding to vascular endothelial growth factor, promoting endothelial cell proliferation, and interacting with stromal proteins by using integrins as a scaffold for cell migration.^17^ It was indicated that tumor cells can promote blood coagulation by interacting with endothelial cells and platelets, and releasing active substances to activate platelets, resulting in increased fibrinogen levels in the blood of cancer patients. In previous studies, high levels of fibrinogen α chain fragments were detected in cholangiocarcinoma,^18^ hepatocellular carcinoma,^19^ and gastric carcinoma.^20^ Klupczynska et al.^21^ combined ZipTip technology with MALDI‐TOF‐MS for the first time to study the serum proteome of patients with NSCLC and healthy controls. It was found that fibrinogen α chain was highly expressed in the patient group compared with the control group. LV et al.^22^ confirmed that fibrinogen α chain was highly expressed in both serum and urine of patients with lung cancer, and speculated that the high expression of fibrinogen α chain might be related to the hypercoagulable state of cancer patients. In this study, fibrinogen α chain showed a high expression level in patients with NSCLC, decreased in the control group, and further decreased after surgery, indicating that fibrinogen *α* chain may play a role in promoting tumor development. Nevertheless, the mechanism of fibrinogen *α* chain in NSCLC is yet unknown. Therefore, the long‐term aim of this study was to expand the number of samples available for further research, as well as to evaluate the protein/peptide expression through immunological methods, so as to identify new tumor markers of lung cancer, and study its molecular mechanism in the process of lung cancer.

Proteomic profiling of serum samples based on the MALDI‐TOF‐MS technology coupled with magnetic beads demonstrated differences in the serum protein expression of NSCLC patients compared with the healthy control group. The GA classification algorithm yielded a discriminative model with features that can distinguish NSCLC patients from healthy controls effectively. Moreover, nano‐LC/ESI‐MS/MS identified fibrinogen *α* chain as potential biomarkers for distinguishing NSCLC, but its final application in clinical screening still needs to be repeatedly verified and further improved by multi‐center large‐sample clinical trials. In conclusion, serum proteome analysis based on MALDI‐TOF‐MS technology is a rapid and accurate platform for the detection of NSCLC. It has great potential for diagnostic applications, screening and regular surveillance in large populations, all of which are crucial for the pandemic control.

## Data Availability

All data generated or analyzed during this study are available from the corresponding author on reasonable request.
